# Understanding the Mode of Action of a Micro-Immunotherapy Formulation: Pre-Clinical Evidence from the Study of 2LEBV^®^ Active Ingredients

**DOI:** 10.3390/life14010102

**Published:** 2024-01-09

**Authors:** Camille Jacques, Flora Marchand, Mathias Chatelais, Adrien Brulefert, Ilaria Floris

**Affiliations:** 1 Pre-Clinical Research Department, Labo’Life France, Pescalis-Les Magnys, 79320 Moncoutant-sur-Sevre, France; ilaria.floris@labolife.com; 2 ProfileHIT, 7 rue du Buisson, 44680 Sainte-Pazanne, France; flora.marchand@profile-hit.com (F.M.); mathias.chatelais@profile-hit.com (M.C.); 3 QIMA Life Sciences, 1 bis rue des Plantes—CS 50011, 86160 Gençay, France; adrien.brulefert@qima.com

**Keywords:** micro-immunotherapy, Epstein–Barr virus, cytokines, immunotherapy, interleukin-2, EBV-associated diseases

## Abstract

Background: Epstein–Barr virus (EBV) is often kept silent and asymptomatic; however, its reactivation induces a chronic and/or recurrent infection that is associated with numerous diseases, including cancer and inflammation-related disorders. As no specific treatment is currently available, the immune factors-based micro-immunotherapy (MI) medicine 2LEBV^®^ could be considered a valuable therapeutic option to sustain the immune system in EBV reactivation. Methods: The present work aimed to investigate, for the first time, the effect of 2LEBV^®^ in several in vitro models of uninfected immune-related cells. Results: 2LEBV^®^ displayed phagocytosis-enhancing capabilities in granulocytes. In human peripheral blood mononuclear cells (PBMCs), it increased the intra- and extra-cellular expression of interleukin (IL)-2. Moreover, it modulated the secretion of other cytokines, increasing IL-4, IL-6, and tumor necrosis factor-α levels or lowering other cytokines levels such as IL-9. Finally, 2LEBV^®^ reduced the expression of human leukocyte antigen (HLA)-II in endothelial cells and macrophages. Conclusions: Although these data are still preliminary and the chosen models do not consider the underlying EBV-reactivation mechanisms, they still provide a better understanding of the mechanisms of action of 2LEBV^®^, both at functional and molecular levels. Furthermore, they open perspectives regarding the potential targets of 2LEBV^®^ in its employment as a therapeutic intervention for EBV-associated diseases.

## 1. Introduction

Identified for the first time in 1964 as the etiologic origin of Burkitt’s lymphoma (BL), the lymphotropic Epstein–Barr virus (EBV) is currently the most ubiquitous of human viruses, as it is thought to infect up to 90% of the adult population around the world [1]. This herpes virus establishes lifelong persistent infections in humans. Although primary infection in childhood remains, most of the time, devoid of clinical symptoms, it can cause infectious mononucleosis in healthy teenagers [2].

Not only is EBV the driving factor for BL, but it has also been reported to be the main oncogene in other lymphoma subtypes, such as diffuse large B-cell lymphoma [3] and Hodgkin’s lymphoma [4], as well as in malignancies of epithelial origin including nasopharyngeal carcinoma [5] and gastric cancers [6]. Moreover, chronic/recurrent EBV infection has been linked to systemic autoimmune diseases [7]. In addition, serological evidence of EBV reactivation was associated with long COVID symptoms, in a cohort of 280 adults with prior severe acute respiratory syndrome (SARS)-CoV-2 infections [8].

As an immune regulators-based low-dose immunotherapy, micro-immunotherapy (MI) could be an interesting therapeutic option in order to prevent and manage EBV-derived chronic inflammatory diseases. MI medicines (MIM) are manufactured as sugar globules, enclosed into capsules, impregnated with active ingredients, and intended to be taken in a fasted state through oromucosal administration. Micro-immunotherapy formulations are developed as complex formulations, combining several ingredients such as human recombinant (hr) cytokines and immune factors in association with nucleic acids preparations, the latter being either under the form of plant-derived total deoxyribonucleic acid (DNA)/ribonucleic acid (RNA) or as specific nucleic acids (SNA^®^) at low doses (LD) and ultra-low doses (ULD). In MIM, the active substances are expressed in centesimal Hahnemannian dilution (CH), and they intend to govern the orientation of a particular biological response towards either an activation or an inhibition, depending on the CH range employed. Indeed, as previously reported, the LD, ranging from 3 CH to 6 CH, were shown to display an immune-boosting effect [9,10]. In contrast, ULD (from 7 CH up to 30 CH), exert a modulatory/inhibitory effect [11,12,13,14,15,16,17,18]. In addition, the efficacy of MI has been documented in various in vitro and in vivo models, from airway allergic diseases and rheumatoid arthritis to cancers.

The treatments available nowadays to manage EBV infections are symptomatic, and no specific antiviral products demonstrate clinical efficacy in patients. Currently, there are four interventional phase IV studies (NCT05039580, NCT04989491, NCT02213068, and NCT04308278), evaluating the efficacy of drugs, therapy, procedures, or medical devices in the context of EBV infection, and one of them is aimed at evaluating the clinical efficacy of MI. In particular, this clinical study assesses the effectiveness of two MIMs (2LEBV^®^ and 2LXFS^®^) in reducing asthenia and other common symptoms of EBV infection.

The MIM 2LEBV^®^ is approved in Belgium by the Federal Agency for Medicines and Health Products (FAMHP) with this authorization number HO-BE517377. This medicine is prescribed as an adjuvant treatment to support the immune system in the case of acute or chronic EBV infection/reactivation, diagnosed by a doctor, and confirmed by biology analysis. However, as far as a clinical trial is still ongoing, no preclinical data are available on 2LEBV^®^, thus the current study focuses on the immunological effects of some of the actives used in 2LEBV^®^ to provide preclinical-based evidence on their possible mode of action.

As it is now well-established that any disturbance of the host immune system can lead to EBV’s reactivation [19,20], an adjuvant treatment able to boost the immune system could help in controlling EBV reactivation, decreasing viral load, and, in turn, reducing the associated symptoms. In order to investigate how 2LEBV^®^ influences the immune responses at the cellular level, we started assessing the effect of the actives from two specific capsules of 2LEBV^®^ in several models of non-infected immune-related human cells. The composition of the two tested capsules, further referred to as 2LEBV^®^-4 and 2LEBV^®^-5, is described in Table 1.

The novelty of the results presented in the current manuscript revolves around the hypothesis that the actives from 2LEBV^®^ could modulate the immune reactions, and they highlighted for the first time the effects of this MIM on several immune-related features, evaluated at functional and at molecular levels. In particular, phagocytosis is an innate immune-related mechanism that can limit the spread of the virus during the early stages of infection by direct elimination of infectious pathogens, the evaluation of this biological process has been included in the readouts measured here. Moreover, as IL-2 constitutes an important mediator of immune activation, and as its emerging role in the crosstalk between innate and adaptive immune responses has been documented [21], a particular emphasis has been placed on the evaluation of the intra-cellular production and the secretion of this cytokine. The rationale behind the choice of the capsules of interest in this study relies on the presence of IL-2 at 7 CH in the formulation of 2LEBV^®^-4. Thus, the effect of this particular capsule has been assessed first on the phagocytosis capabilities of human granulocytes. In addition, as concanavalin A (ConcA) is a known T-cell stimulator and inducer of IL-2 secretion [22], an in-depth analysis of the effect of 2LEBV^®^-4 on the cytokine secretion of unstimulated and ConcA-stimulated peripheral blood mononuclear cells (PBMCs) was also conducted. Namely, the secretion of IL-2 itself, as well as the one of IL-4, -5, -6, -9, -17F, -22, and tumor necrosis factor α (TNF-α) were appraised by enzyme-linked immunosorbent assay (ELISA) assay in those cells.

Having observed an increased IL-2 secretion in the cells treated with the medicine, and in order to better understand how 2LEBV^®^-4 could affect IL-2 generation, an intracellular detection of IL-2 production was conducted by flow cytometry, as this technology also allowed to discriminate its cellular origin amongst the cells that make up the PBMCs. In this attempt, the CD4^+^ and CD8^+^ T-cells, the B-cells, the monocytes, and the natural killer (NK) cells were analyzed. In addition, the effect of 2LEBV^®^-4 on the proliferation of these sub-populations was also measured.

Another particularity of 2LEBV^®^ relies on the presence of the SNA^®^-human leukocytes antigen (HLA)-II at 18 CH in 2LEBV^®^-5 capsule. Considering that the ULD used in MI are intended to modulate/inhibit the expression of their specific targets, if those are upregulated and dysregulated in some pathological contexts [11,12,13,14,15,16,17,18], the second part of the current study aimed at assessing the effects of 2LEBV^®^-5 on the expression of HLA-II. Two models of immune-related cells were employed, namely, human umbilical vein endothelial cells (HUVEC) and PBMC-derived M1 macrophages. In these experimental models, the basal HLA-II levels were increased by stimulations with interferon γ (IFN-γ) and lipopolysaccharide (LPS), respectively, to test the effects of 2LEBV^®^-5 capsule under proper HLA-II-upregulated condition.

The overall data of this study, whereas limited to the cellular immunological effects of only two capsules, evaluated in models that are not characterized by a bona fide reactivation of EBV provide for the first time preclinical-based evidence of the mechanisms of action of 2LEBV^®^, both at functional and molecular levels, and complement the body of knowledge about MI and the rationale behind the use of LD and ULD in their formulations.

## 2. Materials and Methods

### 2.1. Tested Items and Experimental Controls

The tested micro-immunotherapy medicine (MIM) 2LEBV^®^ is a homeopathic medicinal product consisting of a sequence of five different capsules containing sucrose–lactose pillules, impregnated with their unique ethanolic preparations of interleukins, plant-extracted DNA and RNA, as well as specific nucleic acids (SNAs^®^), to be taken repeatedly. The medicine is presented in sequential treatment and is intended to be taken orally, according to the order indicated in the blister. The composition of the two tested capsules (2LEBV^®^-4 and 2LEBV^®^-5) is detailed in Table 1. The 2LEBV^®^ and the vehicle (Veh.) capsules were both manufactured by Labo’Life España, avenida des Raiguer, 7, 07330 Consell–Mallorca, Spain, as previously described [9,10], and have been provided for investigational purposes. The complete composition of 2LEBV^®^ is as follows: human recombinant (hr)-interleukin (IL)-1β at 7 or 10 centesimal Hahnemannian (CH) (7–10 CH); hr-IL-2 (7–10 CH), DNA, and RNA extracted from plants (8–10 CH), SNA^®^-EBV (10–18 CH), and SNA^®^-HLA-II (10–18 CH).

Previous publications have already described how the Veh. capsules are produced in order to provide a suitable control for pre-clinical research [9,10,11,12,13]. In the current study, 2LEBV^®^ or Veh. capsules were freshly diluted in 100 mL or 50 mL of culture medium to reach the final sucrose–lactose concentration of 11- or 22-mM, respectively, and this procedure was performed in all experiments.

### 2.2. Evaluation of the Phagocytosis Capabilities of Human Granulocytes

Granulocytes were isolated from the total peripheral blood of one healthy donor after Ficoll^®^ gradient separation. The cells were grown in Roswell Park Memorial Institute Medium (RPMI)-1640 supplemented with L-glutamine 2 mM, Penicillin 100 U/mL-Streptomycin 100 μg/mL, and Bovine Serum Albumin (BSA) 0.1% at 37 °C and 5% CO_2_. The experiment was performed in 96-well plates; briefly, the cells from one donor were treated either with the Veh. or 2LEBV^®^-4 (both at 22 mM), and these treatments were pre-incubated with the cells for 10 min at 37 °C. Fluorescent beads (Molecular Probes^TM^ (Invitrogene Life Technologies, Eugene, OR, USA) FluoSpheres^TM^ (Invitrogen Life Technologies, Eugene, OR, USA) Carboxylate-Modified Microspheres, 1.0 μm, yellow-green fluorescent 505/515) were then added and incubated with the cells for the next 45 min. Untreated non-bead-incubated cells were used as a negative control. All the conditions were performed in triplicate. After the incubation period, cells were rinsed in phosphate-buffered saline (PBS)/(BSA) 0.1% and centrifuged. Acquisitions were realized with 10,000 cells/replicate on a BD FACSVerse^TM^ (BD Biosciences, Franklin Lakes, NJ, USA) cytometer. Regarding the fact that fluorescence increase in the fluorescein isothiocyanate (FITC) channel is proportional to the phagocytosed beads (emission beads wavelength = 515 nm), the results were first expressed as FITC-positive percentages, then expressed in percentage of the Veh.-treated cells, the latter being set as 100%.

### 2.3. Assessment of the Cytokine-Secretion Profile in Peripheral Blood Mononuclear Cells

Two healthy volunteers per stimulation condition (#1 and #2) were enrolled by the Blood Bank Center (Etablissement Français du Sang, [EFS], Pays de Loire, France). Peripheral blood mononuclear cells (PBMCs) were isolated from the blood of these donors and cultivated in RPMI 1640, added with 2% human serum, non-essential amino acids (1 mM), pyruvate (1 mM), L-glutamine (2 mM) and 4-(2-hydroxyethyl)-1-piperazineethanesulfonic acid (HEPES) buffer (10 mM). The cells were plated at 200,000 cells/well and treated with either the Veh. or 2LEBV^®^-4 at the final concentration of 11 mM sucrose–lactose, added or not with 5 µg/mL concanavalin A (ConcA) during 48 h. The secretion levels of eight cytokines (IL-2, IL-4, IL-5, IL-6, IL-9, IL-17F, IL-22, and TNF-α) were evaluated by ELISA assay, within the supernatant (SN) in each treatment condition.

### 2.4. Evaluation of the Intracellular Expression of IL-2 and the Viability of Human Peripheral Blood Mononuclear Cells

Two healthy volunteers (#1 and #2) were enrolled by the Blood Bank Center (Etablissement Français du Sang, [EFS], Pays de Loire, France). PBMCs were isolated from the blood of these donors and cultivated in RPMI 1640 added with 2% human serum, non-essential amino acids (1 mM), pyruvate (1 mM), L-glutamine (2 mM), and HEPES buffer (10 mM). The cells were plated at 200,000 cells/well and treated with either the Veh., or 2LEBV^®^-4 at the final concentration of 11 mM sucrose–lactose and 5 µg/mL ConcA during 48 h. The cells were then harvested and immune-stained with fluorescent antibodies in order to analyze the intracellular IL-2 content amongst the PBMC sub-populations, labeled as followed: CD4^+^ T-cells: CD3^high^, CD11b^neg^, CD4^high^, CD8^neg^, CD19^neg^, CD56^neg^, CD14^neg^, CD16^neg^, SSC^low^; CD8^+^ T-cells: CD3^high^, CD11b^neg^, CD4^neg^, CD8^high^, CD19^neg^, CD56^neg^, CD14^neg^, CD16^neg^, SSC^low^; B-cells: CD3^neg^, CD11b^neg^, CD4^neg^, CD8^neg^, CD19^high^, CD56^neg^, CD14^neg^, CD16^neg^, SSC^low^; conventional monocytes (C. mono): CD3^neg^, CD11b^high^, CD4^neg^, CD8^neg^, CD19^neg^, CD56^neg^, CD14^high^, CD16^neg^, SSC^low^; non-conventional monocytes (N.C. mono): CD3^neg^, CD11b^high^, CD4^neg^, CD8^neg^, CD19^neg^, CD56^neg^, CD14^neg^, CD16^high^, SSC^low^; natural killer (NK): CD3^neg^, CD11b^neg^, CD4^neg^, CD8^low^, CD19^neg^, CD56^high^, CD14^neg^, CD16^dim^, SSC^low^. The cells were analyzed by flow cytometry on a BD FACS Canto II, configuration 4/2/2.

### 2.5. Evaluation of the Expression of Human Leukocyte Antigen-II

#### 2.5.1. In HUVEC Cells

Human umbilical vein endothelial cells (HUVEC) cells (passage 5, pool from 6 donors, ref: C-12203, Promocell, Heidelberg, Germany), were plated at the density of 5000 cells/well on Day 0 (D0), in a 96-well plate, in endothelial cell growth medium (ref: C-12010, Promocell) added with 2% fetal bovine serum (FBS) (ref: #P30-3302, batch number P170201, PAN-Biotech GmbH, Aidenbach, Germany). The cells were treated on D4 with recombinant human IFN-γ 20 ng/mL (ref: #300-02, batch number #091927, PeproTech, Inc., by Thermo Fisher Scientific, Waltham, MA, USA), w/wo either the Veh., or 2LEBV^®^-5, at the final sucrose–lactose concentration of 11 mM, for the next two days. At the end of the incubation period, the cells were harvested, immune-stained with anti-HLA-II antibody (ref: #307622, BioLegend, San Diego, CA, USA), fixed, and the HLA-DR expression level was assessed by flow cytometry, on a BD FACS Canto II, configuration 4/2/2.

#### 2.5.2. In Human Peripheral Blood Mononuclear Cell-Derived M1-Macrophages

Healthy volunteers were enrolled by the Blood Bank Center (EFS, Pays de Loire, France). All blood samples were approved by the Ethics committee of the EFS Blood Bank Center, with written informed consent obtained for all the donors, following the Declaration of Helsinki. Briefly, PBMCs were isolated from 2 donors (#1 and #2), plated in 96-well plate at the density of 500,000 cells/well, and cultivated in complete RPMI medium (ref: #P04-17500, batch number #7131121, PAN-Biotech GmbH), added with 2% decomplemented human AB serum (ref: #H4522, batch number #SLCC1483, Sigma-Aldrich, Saint-Louis, MO, USA). On D1, the cells were concomitantly treated with recombinant human IFN-γ 20 ng/mL (PeproTech), w/wo either the Veh. or 2LEBV^®^-5, at the final sucrose–lactose concentration of 11 mM. On D4, the media/treatments were renewed, and the cells were challenged with 100 ng/mL lipopolysaccharide (LPS) (ref: #L6529, batch number #059M4103V, Sigma). On D7, the cells were harvested, immune-stained with anti-HLA-DP antibody (ref: #566825, BD Biosciences, Franklin Lakes, NJ, USA), fixed, and the viability as well as the HLA-DP expression levels were assessed by flow cytometry.

### 2.6. Statistical Analysis

The graphs in the figures were performed with GraphPad Prism, Version 10.0.3.275 for Windows (GraphPad Software Inc., San Diego, CA, USA, updated on 3 October 2023). Authors have followed the recent recommendations of D.L. Vaux that encourage performing descriptive statistics instead of making statistical inferences when the number of independent values is small [23]. Indeed, no statistical inference has been performed to analyze the results of the studies presented here. If the results are derived from only one, two, or three (*n* = 1, or *n* = 2, or *n* = 3) experiment(s), it is always better to include a full dataset, plotting data points and letting the readers interpret the data for themselves, rather than drawing statistical inferences, showing *p* values, standard error of the mean (S.E.M.), or results that are not representative.

## 3. Results

### 3.1. Actives from 2LEBV^®^ Enhance the Phagocytosis Capabilities of Human Granulocytes

**Figure 1:**
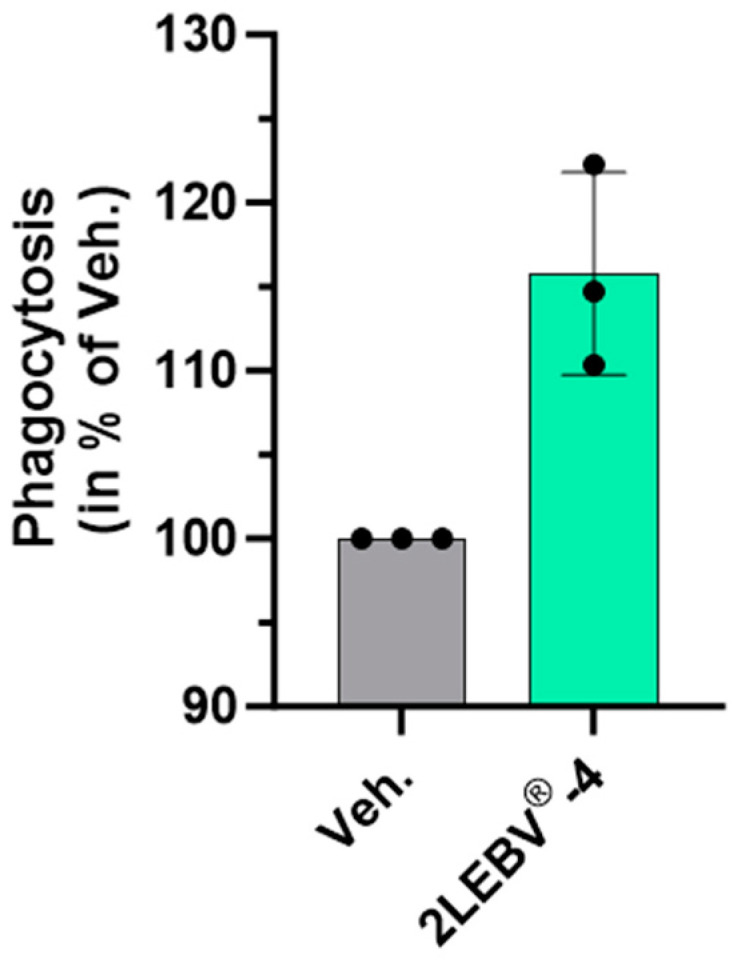
2LEBV^®^-4 increases the phagocytosis capabilities of human granulocytes. Human granulocytes were used to assess the effects of the medicine on the phagocytosis. The cells were plated, and fluorescent beads were added to the culture medium for an additional 45 min, and the percentage of fluorescein isothiocyanate (FITC)-positive cells was appraised by flow cytometry. Human granulocytes were treated with either the vehicle (Veh.) or 2LEBV^®^-4. Each condition was performed in triplicate. Each histogram represents the mean ± standard deviation (S.D.) of fluorescence as a percentage of the Veh.-treated conditions, set at 100%.

In order to assess the effect of the medicine on the phagocytic capabilities, 2LEBV^®^-4 was first investigated on this key functional aspect of the immune system. Thus, its effect was appraised on the phagocytosis capability of human granulocytes. Briefly, human granulocytes were pre-incubated for 10 min with either the vehicle (Veh.), used as a control (see Section 2.2, in Materials and Methods), or with 2LEBV^®^-4. Fluorescein isothiocyanate (FITC)-fluorescent beads were then added to the culture medium for an additional 45 min, and the phagocytosis capabilities of these cells were assessed by flow cytometry, as a measure of the percentage of FITC-positive cells. 2LEBV^®^-4 displayed an immune stimulant effect by slightly enhancing the phagocytosis capabilities of human granulocytes by about 15% (Figure 1), under the tested experimental conditions.

Even if additional tests should confirm these data, these results suggest that within the complete sequence of the 2LEBV^®^ medicine, at least 2LEBV^®^-4 could contribute to its granulocytes-related phagocytosis-enhancing properties, and may sustain the host immune responses under viral infections.

### 3.2. Actives from 2LEBV^®^ Enhance the Secretion of Interleukin-2 in Unstimulated and Concanavalin A-Stimulated Human Peripheral Blood Mononuclear Cells

**Figure 2:**
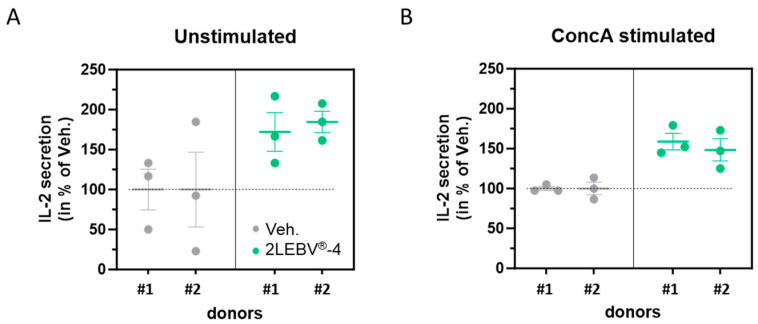
2LEBV^®^-4 enhances the secretion of IL-2 in unstimulated and concanavalin A (ConcA)-stimulated human PBMCs. PBMCs from two donors (#1 and #2) were retrieved and incubated for 48 h in the presence of either the vehicle (Veh.; grey dots), or 2LEBV^®^-4 (green dots), (**A**) in basal culture conditions (unstimulated), or (**B**) concomitantly with 5 µg/mL ConcA (ConcA stimulated). For each donor, the IL-2 secretion was appraised in the supernatants (SN) by ELISA assay. The results are expressed as the mean ± standard error of the mean (S.E.M.) of *n* = 3 measures for each donor and expressed as a percentage of the values obtained in the Veh.-treated conditions (set as 100%). The dotted lines are drawn to highlight the effect of 2LEBV^®^-4 compared with the Veh.

The efficiency of the immune system, and in particular, the T-cell responses, are one of the significant parameters that intervene in the control of EBV during the execution of its latency program or its reactivation [24]. As IL-2 is a key regulator of T-cell metabolic programs [25], we wanted to assess the IL-2 secretion-enhancing capability of the actives from 2LEBV^®^. The secretion levels of IL-2 were thus appraised by ELISA assay in a model of human PBMCs after a 48 h incubation period, in the presence of either the Veh. or 2LEBV^®^-4, with or without a pro-inflammatory stimulus. To that end, we used 5 µg/mL of ConcA, a known T-cell stimulator [22] and inducer of IL-2 secretion, in order to provide such an inflammatory environment to the cells (Supplementary Figure S1A). 2LEBV^®^-4 was tested because it contains IL-2 (7 CH) in its composition (Table 1). As illustrated in Figure 2A, 2LEBV^®^-4 induced a rough increase of about 75% in the IL-2 secretion within the supernatants (SN) in the two assessed donors compared with the Veh. in unstimulated conditions. In addition, it also triggered an overall increase of about 50% in IL-2 secretion in ConcA-stimulated conditions (Figure 2B). Overall, these data could highlight the immune-boosting effect of 2LEBV^®^-4 in enhancing the IL-2 secretion in both unstimulated and ConcA-stimulated PBMCs.

### 3.3. Actives from 2LEBV^®^ Modulate the Secretion of Several Cytokines in Unstimulated and Concanavalin A-Stimulated Human Peripheral Blood Mononuclear Cells

**Figure 3:**
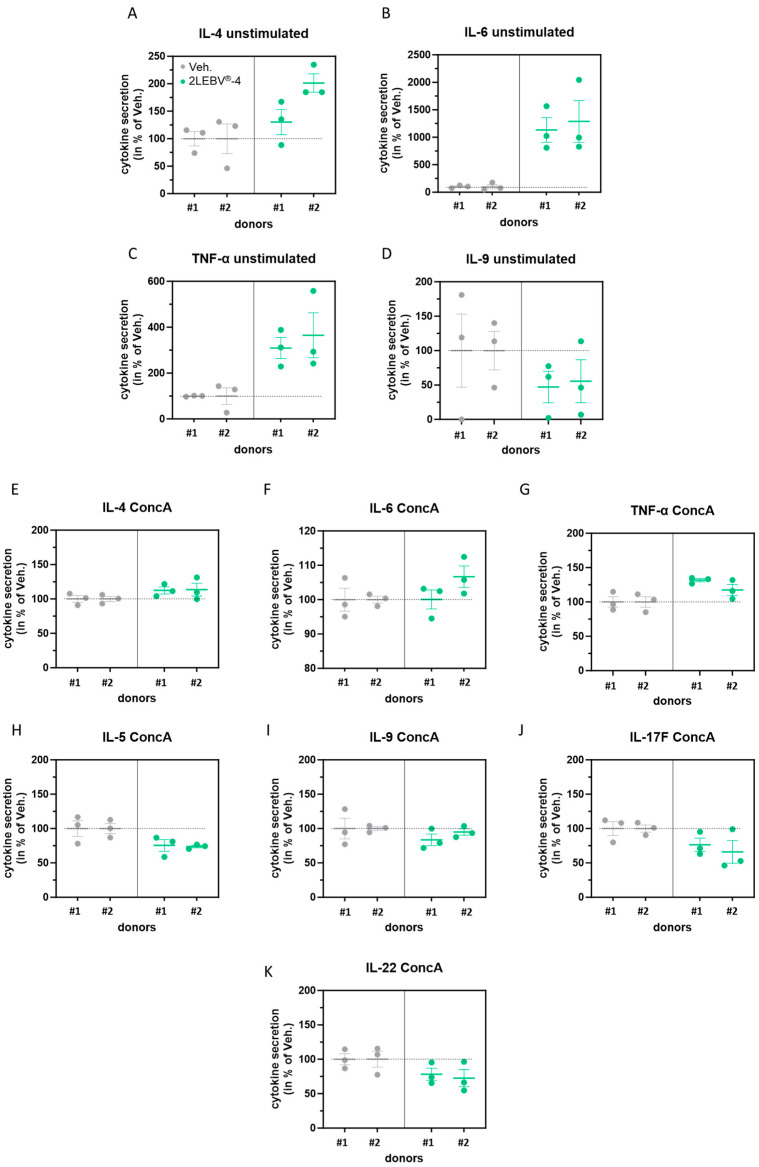
2LEBV^®^-4 modulates the secretion of several cytokines, both in (**A**–**D**) unstimulated and (**E**–**K**) concanavalin A (ConcA)-stimulated human PBMCs. For each stimulatory condition, PBMCs from two donors (#1 and #2) were retrieved and incubated for 48 h in the presence of either the vehicle (Veh.; grey dots) or 2LEBV^®^-4 (green dots), in normal culture conditions (unstimulated) or concomitantly with 5 µg/mL ConcA (ConcA) as an inflammatory stimulus. Thus, ELISA assay appraised the secretion of IL-4, IL-5, IL-6, IL-9, IL-17F, IL-22, and TNF-α in the SN. The results are presented as the mean ± S.E.M. of *n* = 3 measures for each donor and expressed as a percentage of the values obtained in the Veh.-treated conditions (set as 100%). The dotted lines are drawn to highlight the effect of 2LEBV^®^-4 compared with the Veh.

IL-2 influences the homeostasis and development of immune cells, as well as their cytokines level expression [25]. Having observed an increased secretion of IL-2 in unstimulated and in ConcA-stimulated PBMCs treated with 2LEBV^®^-4, we have also assessed the secretion of TNF-α, IL-4, IL-5, IL-6, IL-9, IL-17F, and IL-22, as these factors are also known to be implicated in the regulation of inflammation. The results presented in Figure 3A–C show that 2LEBV^®^-4 could enhance the secretion of IL-4, IL-6, and TNF-α in unstimulated PBMCs compared with the Veh. With mean values of 1132% for donor #1 and 1288% for donor #2 compared with the 100% respective responses to the Veh., 2LEBV^®^-4 drastically increased the production of IL-6 by the PBMCs. In addition, this capsule also reduced the secretion of IL-9 in the two tested donors (Figure 3D). The cytokine dosage in the SN was then appraised in the ConcA-stimulation condition alone, in which the levels of all the assessed cytokines were strongly increased (Supplementary Figure S1B–H), suggesting that this model allows the marked upregulation of all tested cytokines. When the cells were treated with 2LEBV^®^-4 under ConcA-stimulated conditions, we also could appreciate the in vitro co-stimulatory effects of the medicine on the tested cytokines. For instance, and as previously observed in the unstimulated state (Figure 3A–D), the TNF-α, IL-4, and IL-6 levels were also increased by 2LEBV^®^-4 when compared to the Veh. (Figure 3E–G) in ConcA stimulation conditions. On the other hand, whereas the level of IL-9 was also reduced in these conditions compared with the Veh., the secretions of three additional cytokines, IL-5, IL-17F, and IL-22, were also found to be decreased (Figure 3H–K), a downward trend that was not obtained in unstimulated conditions (Supplementary Figure S2). A table summarizing the overall results is available in the supplementary section (Supplementary Table S1). Although extremely cautious about those conclusions, these first pieces of evidence would suggest that, in an unstimulated environment and in a ConcA-stimulated context, 2LEBV^®^-4 could favor the upregulation of cytokines such as IL-4, produced by CD4^+^ T-cell helper-2 (Th2) and, at the same time, the increase in cytokines like TNF-α, which are secreted by Th1 effector cells, activating both types of immune response. In addition, it is possible that the concomitant decrease in IL-9, IL-17F, and IL-22, observed as a consequence of 2LEBV^®^-4 treatment within the ConcA stimulatory context, may imply a reduction in the Th17-type immune response, as these cytokines are often produced by Th17 cells [26,27].

### 3.4. Actives from 2LEBV^®^ Enhance the Intracellular Production of Interleukin-2 in a Wide Range of Concanavalin A-Stimulated Human Peripheral Blood Mononuclear Cell Sub-Populations

**Figure 4:**
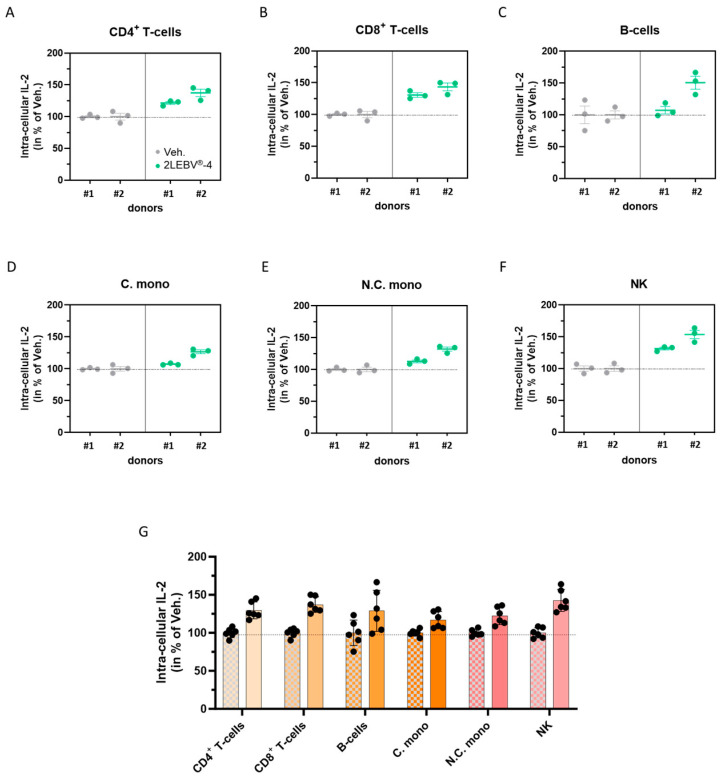
2LEBV^®^-4 enhances the intracellular production of IL-2 in a wide range of concanavalin A (ConcA)-stimulated human PBMC sub-populations. PBMCs from two donors (#1 and #2) were retrieved and incubated for 48 h in the presence of either the vehicle (Veh.; grey dots) or 2LEBV^®^-4 (green dots), concomitantly with 5 µg/mL ConcA. (**A**–**F**) The intracellular production of IL-2 was appraised by flow cytometry within each of the following PBMC sub-populations: the CD4^+^ T-cells, the CD8^+^ T-cells, the B-cells, the conventional monocytes (C. mono), the non-conventional monocytes (N.C. mono), and the natural killer (NK) cells. All the results are presented as the mean ± S.E.M. of *n* = 3 measures for each donor and expressed as a percentage of the values obtained in the Veh.-treated conditions (set as 100%). (**G**) The intracellular levels of IL-2 are illustrated for each of the assessed PBMC sub-populations, the data obtained from the two donors being pooled together. For each sub-population, the grey-squared histograms represent the results for the Veh. condition, used as a control. The dotted lines are drawn to highlight the effect of 2LEBV^®^-4 compared with the Veh.

The second paragraph of the results (Section 2.2) concluded that 2LEBV^®^-4 increased the secretion of IL-2 in ConcA-stimulated human PBMCs (Figure 2B). To deeply investigate the cellular origin of this IL-2 secretion, the intracellular production of IL-2 was evaluated by immune-staining and cytometry analysis in several PBMC sub-populations, under the same treatment conditions. Namely, Figure 4A–G displays the intracellular levels of IL-2 within the CD4^+^ T-cells, the CD8^+^ T-cells, the B-cells, the conventional monocytes (C. mono; characterized by the CD14^high^/CD16^neg^ markers), the non-conventional monocytes (N.C. mono; characterized by the CD14^neg^/CD16^high^ markers), and the natural killer (NK) cells. Overall, the two analyzed donors responded similarly, as 2LEBV^®^-4 increased the intracellular production of IL-2 in all of the assessed cell sub-populations compared to the Veh. With a global increase of about 42% in its intracellular IL-2 production, the NK sub-population was the one in which the highest response was observed (Figure 4G), followed by the CD8^+^ T-cells (about 37% increase). In addition, it could be noticed that the CD4^+^ T-cells and the B-cells were responding almost equally to the 2LEBV^®^-4 treatment (with about a 30% increase). Overall, these results illustrate that, in our experimental setting, whereas all the analyzed immune cells within the PBMCs population displayed an increase in their intracellular production of IL-2 when treated with 2LEBV^®^-4, the NK cells and the CD8^+^ T-cells seemed to be the main producers.

### 3.5. Actives from 2LEBV^®^ Reduce the Overall Proliferation of Several Concanavalin A-Stimulated Human Peripheral Blood Mononuclear Cell Sub-Populations

**Figure 5:**
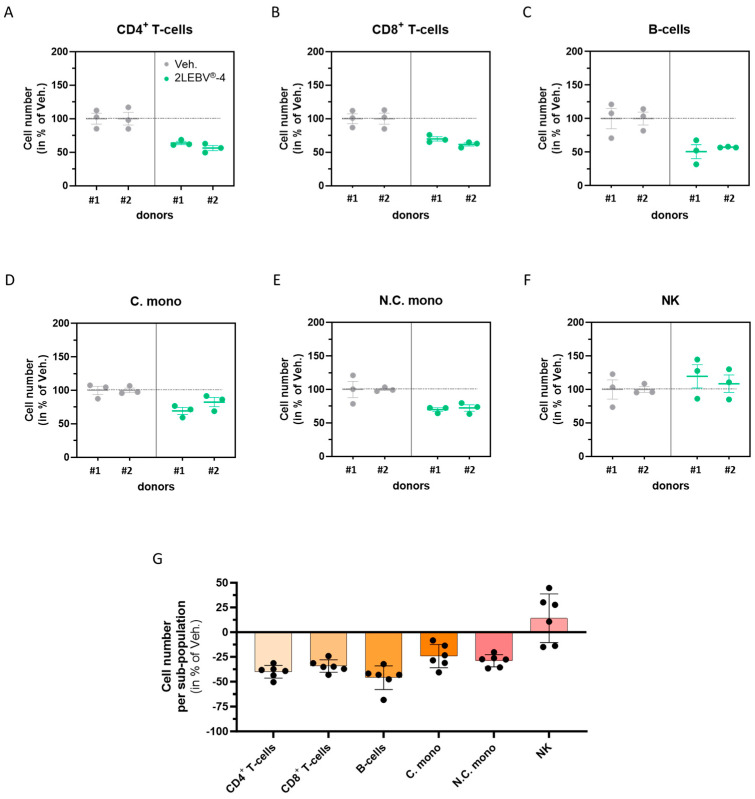
2LEBV^®^-4 reduced the overall proliferation of concanavalin A (ConcA)-stimulated human PBMC sub-populations. PBMCs from two donors (#1 and #2) were retrieved and incubated for 48 h in the presence of either the vehicle (Veh.; grey dots) or 2LEBV^®^-4 (green dots), concomitantly with 5 µg/mL ConcA. (**A**–**F**) For each donor, the cell proliferation was appraised by flow cytometry within each of the following PBMC sub-populations: the CD4^+^ T-cells, the CD8^+^ T-cells, the B-cells, the conventional monocytes (C. mono), the non-conventional monocytes (N.C. mono), and the natural killer (NK) cells. All the results are presented as the mean ± S.E.M. of *n* = 3 measures for each donor and expressed as a percentage of the values obtained in the Veh.-treated conditions (set as 100%). (**G**) The cell proliferation is illustrated for each one of the assessed PBMC sub-populations, with the data obtained from the two donors being pooled together. In panels (**A**–**F**), the dotted lines are drawn to highlight the effect of 2LEBV^®^-4 compared with the Veh.

Interleukin-2 is not only a crucial immunomodulatory cytokine that controls the secretion of multiple other cytokines and immune factors but also has a valuable role in immune cell proliferation [28]. Having found that the actives from 2LEBV^®^-4 enhanced the secretion of IL-2, we also wanted to assess its effect on the proliferation of the ConcA-stimulated PBMC sub-populations (Figure 5A–F). Surprisingly, in these experimental conditions, the cell proliferation was reduced by 2LEBV^®^-4 treatment compared with the Veh. in all the analyzed sub-populations (Figure 5A–E), except in the NK cells, in which the cell number was increased by about 20% in donor #1 and by about 10% in donor #2 (Figure 5F). The proliferation of the B-cells was the most affected by the tested 2LEBV^®^ capsule, as the overall proliferation of this ConcA-stimulated sub-population was reduced by almost 50% compared with the Veh. (Figure 5G). In addition, 2LEBV^®^-4 reduced the proliferation of the CD4^+^ and CD8^+^ T-cells by about 35–40% and one of the C. and N.C. monocytes by about 25%. These results suggest that 2LEBV^®^-4 could display an anti-proliferative effect on the CD4^+^ T-cells, CD8^+^ T-cells, B-cells, and monocytes from ConcA-stimulated PBMCs.

### 3.6. Actives from 2LEBV^®^ Reduce the Expression of Human Leukocyte Antigen-II in Two Models of Human Immune-Related Cells

As human leukocyte antigen (HLA)-II is reported to be one of the receptors participating in EBV’s entry into its target cells [29,30,31] and one of the receptors that can contribute to immune cells’ anergy [32], we wanted to assess the effect of 2LEBV^®^-5, which contains SNA^®^-HLA-II at 18 CH, on the expression levels of HLA-II. Two different human cell types and cellular models were thus employed: (i) HUVEC cells and (ii) human M1-macrophages derived from PBMCs.

**Figure 6:**
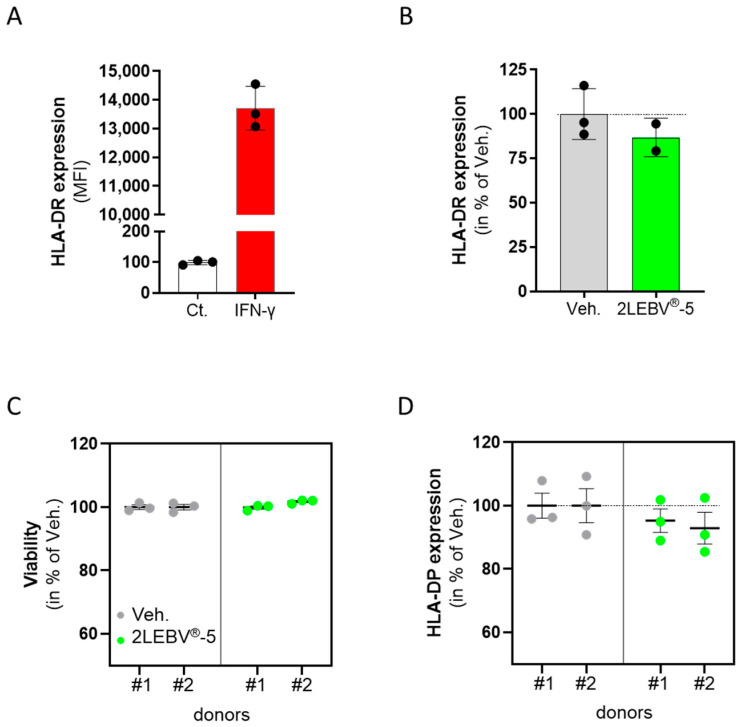
2LEBV^®^-5 slightly reduced the expression of HLA-II in human endothelial cells and in Peripheral Blood Mononuclear Cell-derived human M1-macrophages. (**A**) The expression level of HLA-DR was assessed by flow cytometry in control (Ct.) and in 20 ng/mL interferon (IFN)-γ treated HUVEC cells (IFN-γ; red histogram). The results are expressed as the mean ± S.D. of the median fluorescence intensity (MFI) for *n* = 3 replicates per condition. (**B**) The expression level of HLA-DR was assessed in IFN-γ-stimulated HUVECs, treated with either the vehicle (Veh.; grey histogram) or 2LEBV^®^-5 (green histogram) for 48 h. The results are expressed in percentage of MFI, the Veh. conditions being set at 100%. (**C**) PBMCs were retrieved from two donors (#1 and #2), and the viability of their lipopolysaccharide (LPS)-stimulated PBMC-derived M1-macrophages has been appraised after six days of treatment in the presence of either the Veh. or 2LEBV^®^-5. (**D**) HLA-DP expression was assessed in the same conditions described in (**C**). In (**C**,**D**), the results are presented in percentage of MFI, the Veh. condition is set at 100%. In panels (**B**,**D**), the dotted lines are drawn to highlight the effect of 2LEBV^®^-5 compared with the Veh.

As the expression of HLA-DR can be induced in HUVECs by IFN-γ stimulation [33,34], these stimulated non-immune cells were chosen as a stable and robust model for a basal HLA-DR expression. Briefly, the cells were treated with 20 ng/mL IFN-γ alone or concomitantly with either the Veh. or 2LEBV^®^-5 for 48 h. The cells were immune-stained, fixed, and analyzed by flow cytometry for HLA-DR expression. As shown in Figure 6A, IFN-γ used as a control condition induced a strong HLA-DR expression compared with the unstimulated control (about 13,700 median fluorescence intensity (MFI) vs. about 100 MFI, respectively). Interestingly, in the presence of 2LEBV^®^-5 (Figure 6B), a decrease of about 15% in HLA-DR expression was noticed in comparison to the Veh. As IFN-γ was reported to solely lead to a slight increase in HLA-DP expression in HUVEC cells [35], and as we were not able to detect any HLA-DP in our experimental conditions, a second model of human PBMC-derived M1-macrophages was considered. In this model, PBMCs from two healthy donors were isolated by CD14^+^ selection and cultivated for six days in the presence of 20 ng/mL IFN-γ, in concomitance with either the Veh. or 2LEBV^®^-5. The media/treatments were renewed on day 4 (D4), and the expression of HLA-DP was triggered by a 24 h incubation with 100 ng/mL LPS. On D7, the M1-macrophages’ viability and their level of HLA-DP expression were appraised by flow cytometry. As presented in Figure 6C, whereas 2LEBV^®^-5 did not impact the cell viability compared with the Veh., such treatment led to a slight downward trend in the expression of HLA-DP, by about 5% in donor #1, and by about 8% in donor #2 (Figure 6D).

Overall, even if those small trends need to be confirmed, these data may suggest that 2LEBV^®^-5 could reduce the expression of HLA-II in IFN-γ-stimulated HUVEC cells and in LPS-stimulated human PBMC-derived M1-macrophages.

## 4. Discussion

The immune system is crucial for organisms to combat the invasion of pathogens like bacteria or viruses and is also necessary for protecting against harmful substances. Vertebrates have evolved innate and adaptive responses within their immune system, working together to provide a robust defense against foreign threats [36,37]. Nonetheless, numerous viruses employ immune suppression as a strategy in order to persist and replicate in their host [38]. Epstein–Barr virus (EBV) does not deviate from this principle and it has been reported that EBV miRNAs efficiently inhibit the release of proinflammatory cytokines, including interleukin (IL)-12, dampen the differentiation process of naive CD4^+^ T-cells into Th1 cells, disrupt the peptide processing and presentation on HLA-II class II, thereby decreasing the activation of cytotoxic EBV-specific CD4^+^ effector T-cells, and impeding the elimination of infected B-cells [39]. In this context, it is paramount to find efficient ways to help the immune cells, either the ones from the innate system or those from the adaptive one, to stimulate their biological responses. Micro-immunotherapy may appear as an interesting alternative within the realm of viral infections, especially since the immune-boosting effects of the 2LEID^®^ medicine have been proven, both in vitro and in vivo, in a mice model of influenza A virus [9]. In the present study, we expand the current knowledge about the immune-stimulatory effects of MI, evaluating the effects of two capsules of another medicine, named 2LEBV^®^, in several models of human immune cells, either in an unstimulated basal state or in concanavalin A (ConcA)-, interferon (IFN)-γ-, or lipopolysaccharide (LPS)-triggered inflammatory conditions.

From the innate immunity side, phagocytosis of infected cells constitutes one of the first steps toward organisms’ defenses against viruses. This particular mechanism has been evaluated by Vistarop et al. in their analysis of EBV infection in a series of pediatric carriers from a developing country [40]. Indeed, they interpreted the fact that they found positive staining for the latency III-associated protein EBNA3A within the cytoplasm of sub-epithelial macrophages as a probable effective phagocytosis of EBV-infected cells. In addition, and interestingly enough, the capability of EBV to infect and replicate in freshly isolated human monocytes has been reported, and such EBV-infected monocytes were found to display a significantly reduced phagocytic activity up to 72 h post-infection [41]. Even though these results were obtained in a model of uninfected and unstimulated cells, and should be confirmed in the context of EBV infection, we were able to show for the first time a slight effect of 2LEBV^®^-4 in enhancing the phagocytosis capabilities of human granulocytes (Figure 1). Thus, although preliminary, interestingly this evidence suggests a potential beneficial effect of 2LEBV^®^ in the clearance of infected cells.

Concerning the immune-enhancing and modulating functions of IL-2 [42], and its implication in antiviral responses [43], we wanted to address if and how much 2LEBV^®^ could modulate the secretion of IL-2. We thus showed that 2LEBV^®^-4 stimulated the secretion of IL-2 (Figure 2), both in unstimulated and in ConcA-stimulated human PBMCs. The magnitude of the effect, compared to the Veh. control is quite important as the treatment has increased the IL-2 secretion by about 75% in unstimulated conditions, and by about 50% in ConcA-stimulated conditions. These results are very encouraging if we take into account the crucial role of IL-2 in the induction of immune responses. Moreover, it is not excluded that the phagocytosis-triggering effect that we observed in human granulocytes may be due, at least partially, to the IL-2 employed within the formulation of the assessed capsule of 2LEBV^®^, as the effect of this cytokine has previously been reported, either alone, in a model of B-cells from *Paralichthys olivaceus* [44], or when fused to granulocyte/macrophage colony-stimulating factor (GM-CSF), in the murine, immature dendritic cell line, DC2.4 [45]. Accordingly, low doses of IL-2 therapies have also already been proven to display immune-boosting effects in the context of viral infections and even in pathologies related to EBV infections. Indeed, according to the work from Molloy et al., the administration of IL-2/anti-IL-2 immune complex had a rapid effect in reducing the persistent viral burden in the lungs of CD4^+^ T-cell-depleted mice infected with murine γ-herpesvirus 68 (MHV-68) [46]. Additionally, in a mouse model of chronic lymphocytic choriomeningitis virus (LCMV) infection, daily administration of low-dose IL-2 resulted in an improvement in the CD8^+^ T-cell responses, as well as a reduction in the presence of inhibitory receptors on CD8^+^ T-cells, that were specific to the virus [47]. Baiocchi et Caligiuri demonstrated that very low doses of polyethylene glycol-modified rh-IL-2 therapy could counteract the immune deficiencies leading to EBV-associated lymphoproliferative diseases by using an in vivo model of severe combined immunodeficiency (SCID) mice reconstituted with human peripheral blood lymphocytes [48]. In an attempt to fully benefit from the immune-stimulating effects of IL-2 while minimizing the adverse effects of the IL-2 therapies, it is good to remember that, in a clinical setting, a low dose of IL-2 displayed effects similar to those of higher dosages on CD4^+^ T-cells, with apparently less toxicity [49], which still encourages to employ a 7–10 CH range in therapy, as it is used in the formulation of 2LEBV^®^.

In addition, not only our results suggested that 2LEBV^®^-4 stimulates the secretion of IL-2 in human ConcA-stimulated PBMCs (Figure 2B), but they also highlighted that, even if an increase in the intracellular level of IL-2 was found in all the analyzed immune cells’ populations (Figure 4), it may be possible that this increased secretion could originate in the main part, from the NK and the CD8^+^ T-cell reservoirs (Figure 4B,F,G). Regarding the NK cell responses to 2LEBV^®^-4, and assuming that the IL-2 production may have been triggered by the IL-2 used at 7 CH in the medicine, our findings are consistent with those of De Sanctis et al., who were likely among the pioneers in showing that stimulating NK cells for 18 h with rh-IL-2 at doses ranging from 10 to 500 IU resulted in a dose-dependent increase in the secretion of this cytokine [50]. However, concerning our results, the highest level of intracellular IL-2 production would indeed have been expected within the CD4^+^ T-cells instead of the CD8^+^ ones (Figure 4G), as a way higher level of IL-2 transcription was reported in CD4^+^ T-cells recovered from normal naive BALB/c mice’s spleen samples compared with the CD8^+^ or even the NK cells [51]. Nonetheless, in living organisms, the fact that CD8^+^ T-cells are exposed to varying levels and durations of IL-2 signals and that those cells harbor unique gene expression programs, it is still unclear how the autocrine and paracrine IL-2 signals influence their metabolism [52], and further studies are still needed to better understand the effect of this specific capsule of 2LEBV^®^ on the above-mentioned immune cell populations.

Apart from IL-2, and concerning the modulation of the cytokine secretion, our results showed that 2LEBV^®^-4 treatment induced a PBMCs’ secretion profile characterized by an increase in the IL-4, IL-6, and TNF-α, associated with a decrease in the secretion of IL-9, in unstimulated conditions (Figure 3A–D). Interestingly, the same pattern was found in ConcA-inflamed conditions, in adjunction with the simultaneous downregulation of three additional cytokines: IL-5, IL-17F, and IL-22 (Figure 3E–K). In particular, with an equivalent average increase of about 12 times its initial level within the two assessed donors in unstimulated conditions, the IL-6 secretion level was drastically enhanced by 2LEBV^®^-4 (Figure 3B).

Interleukin-6 is one of the key cytokines involved in the host’s immune response during viral infection [53]. The use of IL-6-deficient mice models has indeed demonstrated the essential role played by this cytokine in the survival of the animals when infected with the influenza virus [54]. Not only did this study show that IL-6 was required for optimal activation of influenza-specific T-cell responses, but also that this cytokine was necessary for controlling viral load, and for limiting the excessive recruitment of damaging inflammatory monocytic cells to the lungs. Moreover, the same mice models were used to highlight the pro-phagocytic properties of IL-6, as influenza virus particles and dead infected cells were more efficiently ingested by the peritoneal macrophages isolated from WT mice than from IL-6^−/−^ mice [55]. The increase in TNF-α that has been induced by 2LEBV^®^-4 (Figure 3C,G) can be seen as an interesting perspective regarding the fact that this factor has also been reported as a cytokine with antiviral activities through its ability to induce the expression of IFN-stimulated genes [56]. Thus, its upregulation could be beneficial in the context of EBV infection. On the other side, the use of TNF-α blockers in patients suffering from severe chronic inflammatory diseases was associated with the activation of latent viral infection, including EBV [57].

Although the nature of these results is preliminary, the concomitant increase in the secretion of IL-4 and TNF-α that we found induced by 2LEBV^®^-4, either in unstimulated or in ConcA-stimulated conditions (Figure 3A,C,E,G), could remind us of Th2- and Th1-oriented responses, respectively. However, it is difficult to conclude about a clear effect of our tested MIM in potentially favoring both the Th1 and the Th2 responses, as the secretion of IL-6 is also enhanced by 2LEBV^®^-4, especially in unstimulated conditions (Figure 3B,F). Indeed, this cytokine has been shown to play a dual role in Th1/Th2 differentiation by promoting Th2 differentiation and simultaneously inhibiting Th1 polarization through two independent molecular mechanisms [58], thus suggesting an effect of our medicine in preferentially orienting the CD4^+^ response towards a Th2 one. Similarly, and subject to the need to corroborate the results, it is interesting to pinpoint that the slight reduction in the levels of IL-5, IL-9, IL-17F, and IL-22 found in the SN from ConcA-stimulated human PBMCs after 2LEBV^®^-4 treatment could suggest that the active ingredients from this capsule may also have induced a decrease in the Th17 cells activation (Figure 3). Of note, this effect could be, at least partially, attributable to the presence of IL-2 in the formulation of the tested medicine, as Laurence et al. showed that impeding IL-2 function through genetic deletion or antibody blockade promoted the differentiation of Th17 cell subset [59]. In line with these results, it is interesting to mention that in mice models and in human studies of viral myocarditis and multiple sclerosis, two conditions in which EBV is involved, the deleterious function of Th17 cells has been delineated [60]. Overall, even if more studies are definitely still needed to draw a conclusion about the CD4^+^-response-polarization effects of 2LEBV^®^-4, avenues for reflection were definitely brought out here.

In this current study, not only have we assessed the cytokine secretion and the origin of the IL-2 secretion within 2LEBV^®^-4-treated PBMCs, but we have also evaluated the effect of the actives from this particular capsule on the proliferation of the PMBC sub-populations. Interestingly, in the context of an existing pre-stimulation, as mimicked in our model by ConcA, this capsule led to reduced proliferation of T-cells, B-cells, and monocytes (Figure 5A–E,G). The results obtained on the latter sub-population are not surprising and could be explained by the fact that monocytes usually respond to cytokines such as IL-2 through functional changes rather than through proliferation [61]. However, IL-2 is commonly seen as a canonical T-cell growth factor, able to induce the clonal expansion of T-cells, under antigen stimulation conditions [52]. In addition to its effects on T-cells, IL-2 also stimulates the proliferation of B-cells [62]. In light of this body of knowledge, our results may appear unpredictable. What we should consider, as a general rule when in vitro data are discussed, is that the immune system, at cellular and organism levels, integrates complex information about the environment. Immune cell proliferation does not follow linear rules and it is controlled by different factors such as the concentration, the expression of the receptors, and finally, but not less importantly, the interaction with other cytokines, which can act synergistically, or not, towards a specific behavior or effect. For example, there are cytokines that can have inhibitory effects on IL-2 induction, such as IL-4 [63]. If we consider that the treatment has induced the upregulation of IL-4, under both tested basal and stimulated conditions, we might have an explanation that can partially elucidate the interpretation of our results.

Interestingly, amongst all the PBMC sub-populations analyzed, the NK cells were the only ones whose proliferation appeared to be stimulated by the actives from 2LEBV^®^-4 (Figure 5F,G). These results could be explained by the capability of IL-2 to stimulate the proliferation of NK cells [64]. In addition, they could also resonate with the extreme sensitivity of those cells to IL-2, taking into account that IL-2 (7 CH) is found in the 2LEBV^®^-4 capsule, and it has previously been reported that only 1.5 ng/mL of IL-2 was sufficient to induce the proliferation of CD56^bright^ NK cells [65]. Moreover, the inhibitory effect of the capsule on the proliferation of the other sub-populations, especially on the T-cells (Figure 5A,B,G), could be linked with previous results from Blattman et al., who reported a dual role of IL-2 therapy on T-cells, depending on their stage of differentiation, in a model of LCMV-infected mice [66]. In this model, these authors showed that, whereas IL-2 was detrimental to the survival of rapidly dividing effector T-cells, the administration of this cytokine was beneficial for chronically infected mice. Additionally, it also appears that not only the timing of IL-2 treatment may be a factor modulating its effects, but its spatial localization, too. Indeed, the vesicular stomatitis virus (VSV) ovalbumin (OVA) in vivo study from D’Souza et al. offered an example of the paradoxical roles of IL-2 in T-cells responses, as a growth factor and as a regulator of cell death, depending on the localization of the CD8^+^ T-cells, either in secondary lymphoid tissues or in non-lymphoid tissues [67]. Nonetheless, it is worth mentioning that, in healthy volunteers, ultra-low doses of IL-2 have already been reported to display immune-modulating functions and to promote the expansion of NK cells [68].

Although this first set of data provides interesting results, potentially suggesting the immune-boosting effects of the actives from 2LEBV^®^, we are perfectly aware that this study has limitations, not only through the sample size restriction but also in the sense that it is difficult to assess the in vitro efficacy of 2LEBV^®^ in the context of an EBV reactivation. Indeed, it is unlikely that the ConcA stimulation that we have chosen to apply to the immune cells could perfectly mimic a reactivation of the EBV virus. However, ConcA is a lectin that can still bind to the carbohydrates present on the cell surface and activate certain cellular signaling pathways such as the just another kinase (JAK)/signal transducers and activator of transcription (STAT)-3, mitogen-activated protein kinase (MAPK), and nuclear factor kappa B (NF-κB) pathways [69]. In the context of pre-existing infection, it is possible that the activation of these signaling pathways could indirectly affect the replication of the EBV virus, but further studies would be needed to understand the specific mechanisms involved. For instance, it is worth mentioning that, in EBV-infected B-cells, the protein kinase C (PKC)θ-p38 MAPK axis was reported to be involved in the promotion of the EBV lytic cycle [70]. In addition, injections of ConcA have been employed in mice, in order to mimic acute hepatitis, with important similarities to immune-mediated hepatitis in humans, such as acute viral hepatitis [71]. In addition, concerning our use of IFN-γ as another immune stimulus in the last part of this study, it can also be contextualized with respect to EBV, as elevated levels of IFN-γ have been found to be associated with high levels of EBV reactivation [72]. Finally, another limitation of this study relies on the lack of awareness regarding the EBV status of the PBMC donors, possibly implying that the analyzed T-cell populations could have been a combination of memory and naïve cells, a question that it would have been interesting to delve into more thoroughly.

In the last results section (Section 3.6), we finally wanted to assess the immune-modulatory effects of 2LEBV^®^-5, and, in particular, its capability to reduce the expression of HLA-DR and HLA-DP, which are used by EBV as infection receptors. Indeed, since EBV has been studied, a growing body of evidence has been collected regarding the potential receptors employed by this virus to infect cells. In particular, Li et al. were one of the pioneers in this field as they reported that EBV used HLA-II as a cofactor for the infection of B-cells [73]. They delineated the importance of HLA-DR in this mechanism as they showed that it was not possible to superinfect cells that failed to express this specific variant. In line with these considerations, the slight effect of 2LEBV^®^-5 in downregulating the expression of HLA-DR in IFN-γ-stimulated endothelial cells (Figure 6B) is quite interesting, especially as primary endothelial cells were also reported to be infectable with EBV in vitro, and able to replicate infectious EBV [74]. As EBV also uses HLA-DP as a co-receptor [30], and as HUVEC cells failed at expressing HLA-DP under IFN-γ stimulation, an alternative model was used, in which PBMC-derived human M1-macrophages were stimulated with LPS. As illustrated in Figure 6C,D, the expression of this HLA-II variant was slightly reduced by 2LEBV^®^-5, without any impact on the cell survival, in the two assessed donors.

Altogether, and even if still relatively preliminary, this body of data could open new directions for future preclinical research. Indeed, the observations made here pave the way for the consideration of complementary alternative models of EBV-naïve or EBV-memory immune cells that could be infected or re-infected in vitro, in order to mimic the conditions of a bona fide infectious mononucleosis or an EBV reactivation. For instance, as the symptoms of this infection are better characterized than the ones from EBV-associated diseases, whose chronicity and numerous other associated factors often blur the onsets, such models could be an interesting tool to bring 2LEBV^®^ research further. The results provided in this study could highlight a dual effect of the assessed capsules of 2LEBV^®^, in both stimulating some features of the immune system and modulating some others at the same time, which, from a clinical standpoint, could help the patients controlling the spread of the virus while avoiding the long-term deleterious effects of over-activated immune cells. To date, the preclinical results provided here are too preliminary and related to only two capsules of the sequential MIM. Further preclinical investigations aimed at assessing the effects of the complete medicine composition would allow a better understanding of its mode of action, and the results of the current clinical trial will be able to provide data about the safety and the efficacy of 2LEBV^®^ in reducing asthenia and other symptoms that are commonly related to EBV infection.

## 5. Conclusions

In conclusion, the current study highlighted interesting properties of two of the capsules making up the entire sequence of the 2LEBV^®^ MI medicine (2LEBV^®^-4 and 2LEBV^®^-5). The slight phagocytosis-enhancing capabilities of 2LEBV^®^-4 were thus revealed in a model of human granulocytes. Moreover, as the immune-stimulatory effects of this capsule were also assessed in unstimulated and ConcA-stimulated PBMCs, our data showed that it increased the intracellular expression and secretion of IL-2, possibly due to the presence of IL-2 (7 CH) in its composition. Our investigations on 2LEBV^®^-4 continued in assessing a larger panel of cytokines, unveiling that this capsule could also modulate the cytokine secretion pattern observed in both Th1 and Th2 polarized T-cells, but also attests to a reduced polarization towards the Th17 profile. Regarding the effect of 2LEBV^®^-4 on the proliferation of the immune cells, it seemed that it could have enhanced the proliferation of the only NK cells amongst the analyzed PBMCs’ subpopulations, under the tested experimental conditions. Finally, our attention focused on 2LEBV^®^-5, which employs the SNA^®^-HLA-II at 18 CH in association with other actives. According to our results, it seems that this capsule has reduced the expression of HLA-DR and HLA-DP in HUVECs and in PBMC-derived human M1 macrophages, respectively.

Altogether, these preliminary experiments provide the first in vitro evidence of the immune effect of two capsules of 2LEBV^®^. Moreover, the dichotomic concept of the LD/ULD that has previously been documented in several published studies seemed to be, once again, corroborated by the current work, regarding the molecular effect of the 2LEBV^®^ capsules on both IL-2 and HLA-II expression.

## Figures and Tables

**Table 1:** Composition of the tested capsules of 2LEBV^®^.

Ingredients (CH)	2LEBV^®^-4	2LEBV^®^-5
hr-IL-1β	10	10
hr-IL-2	**7**	10
DNA	10	**8**
RNA	10	10
SNA^®^-EBV	10	10
SNA^®^-HLA-II	10	**18**

CH: centesimal Hahnemannian dilution; EBV: Epstein–Barr virus; HLA: human leukocytes antigen; hr: human recombinant; IL: interleukin; SNA^®^: specific nucleic acid.

## Data Availability

The data of the current study are available from the corresponding author on reasonable request.

## Supplementary Materials

The following supporting information can be downloaded at: https://www.mdpi.com/article/10.3390/life14010102/s1, Supplementary Figure S1. Concanavalin A induces the secretion of a panel of cytokines when compared with the control unstimulated conditions, in a model of human PBMCs. Supplementary Figure S2. 2LEBV^®^-4 modulates the secretion of several cytokines in unstimulated human PBMCs. Supplementary Table S1. Effect of 2LEBV^®^-4 on cytokine secretion, appraised in unstimulated or in concanavalin A (Con-A)-stimulated human peripheral blood mononuclear cells (PBMCs).

## Abbreviations

BL: Burkitt’s lymphoma; BSA: bovine serum albumin; C. mono: conventional monocytes; ConcA: concanavalin A; DNA: deoxyribonucleic acid; EBV: Epstein–Barr virus; EFS: Etablissement Français du Sang; ELISA: enzyme-linked immunosorbent assay; FAMHP: Federal Agency for Medicines and Health Products; FBS: fetal bovine serum; FITC: fluorescein isothiocyanate; fMLP: N-formyl-methionyl-leucyl-phenylalanine; GM-CSF: granulocyte/macrophage colony stimulating factor; HEPES: 4-(2-hydroxyethyl)-1-piperazineethanesulfonic acid; HLA: human leukocyte antigen; hr: human recombinant; HUVEC: human umbilical vein endothelial cells; IFN-γ: interferon γ; IL: interleukin; JAK: just another kinase; LCMV: lymphocytic choriomeningitis virus; LD: low doses; LPS: lipopolysaccharide; MAPK: mitogen-activated protein kinase; MFI: median fluorescence intensity; MHV-68: murine γ-herpesvirus 68; MI: micro-immunotherapy; MIM: micro-immunotherapy medicine; N.C. mono: non-conventional monocytes; NF-κB: nuclear factor kappa B; NK: natural killer; PBMCs: peripheral blood mononuclear cells; PBS: phosphate-buffered saline; PKC: protein kinase C; RNA: ribonucleic acid; RPMI: Roswell Park Memorial Institute; SARS: severe acute respiratory syndrome; SCID: severe combined immunodeficiency; S.D.: standard deviation; S.E.M.: standard error of the mean; SN: supernatant; SNA^®^: specific nucleic acid; STAT: signal transducers and activator of transcription; TNF-α: tumor necrosis factor α; ULD: ultra-low doses; Veh.: vehicle. VSV: vesicular stomatitis virus; OVA: ovalbumin.
