# Supplementary material for: Understanding the Mode of Action of a Micro-Immunotherapy Formulation: Pre-Clinical Evidence from the Study of 2LEBV® Active Ingredients
Source: Life (Basel). 2024 Jan 9;14(1):102. doi: 10.3390/life14010102 (PMC10821216; doi:10.3390/life14010102)
Supplement: Supplementary file 1 [file life-14-00102-s001.zip › life-2720072-supplementary.pdf]

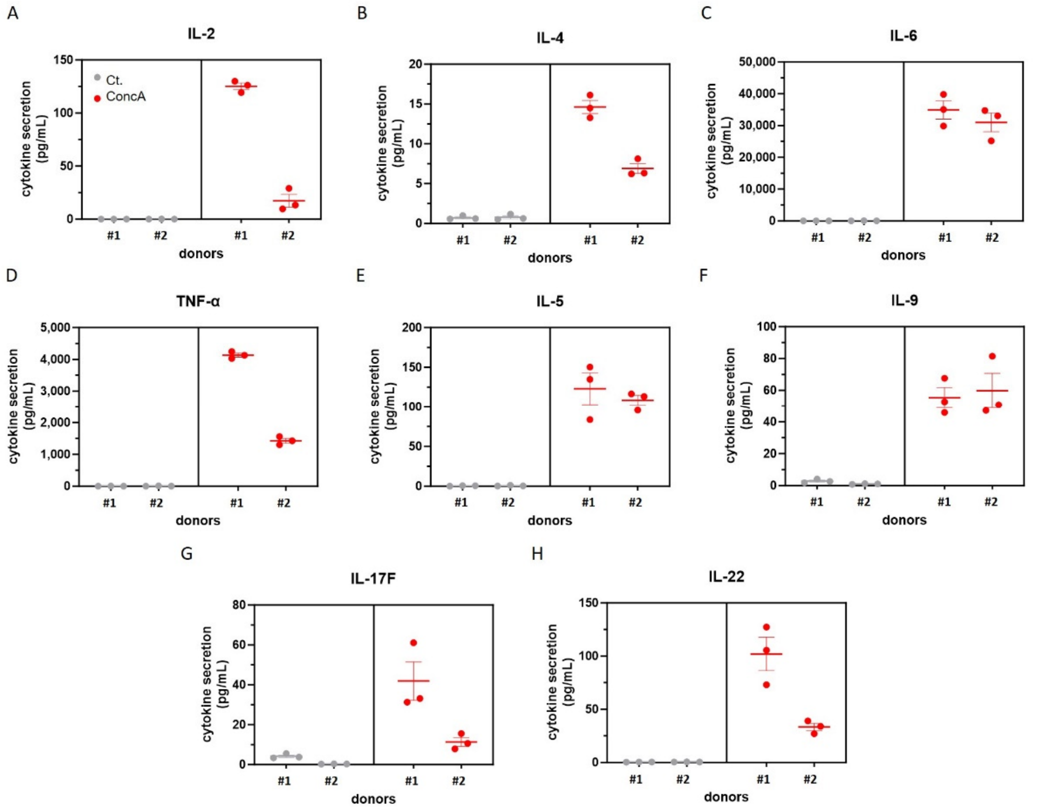

Supplementary Figure S1. Concanavalin A induces the secretion of a panel of cytokines when compared with the control unstimulated conditions, in a model of human PBMCs. PBMCs from two donors (#1 and #2) were retrieved and left untreated (control, Ct., grey dots) or were stimulated for 48 hours with 5 μg/mL ConcA (ConcA, red dots). (**A-H**) For each donor, the secretion of IL-2, IL-4, IL-6, TNF-α, IL-5, IL-9, IL-17F, and IL-22 was appraised in the SN, by ELISA assay. The results are expressed as the mean ± S.E.M. of n = 3 measures for each donor.

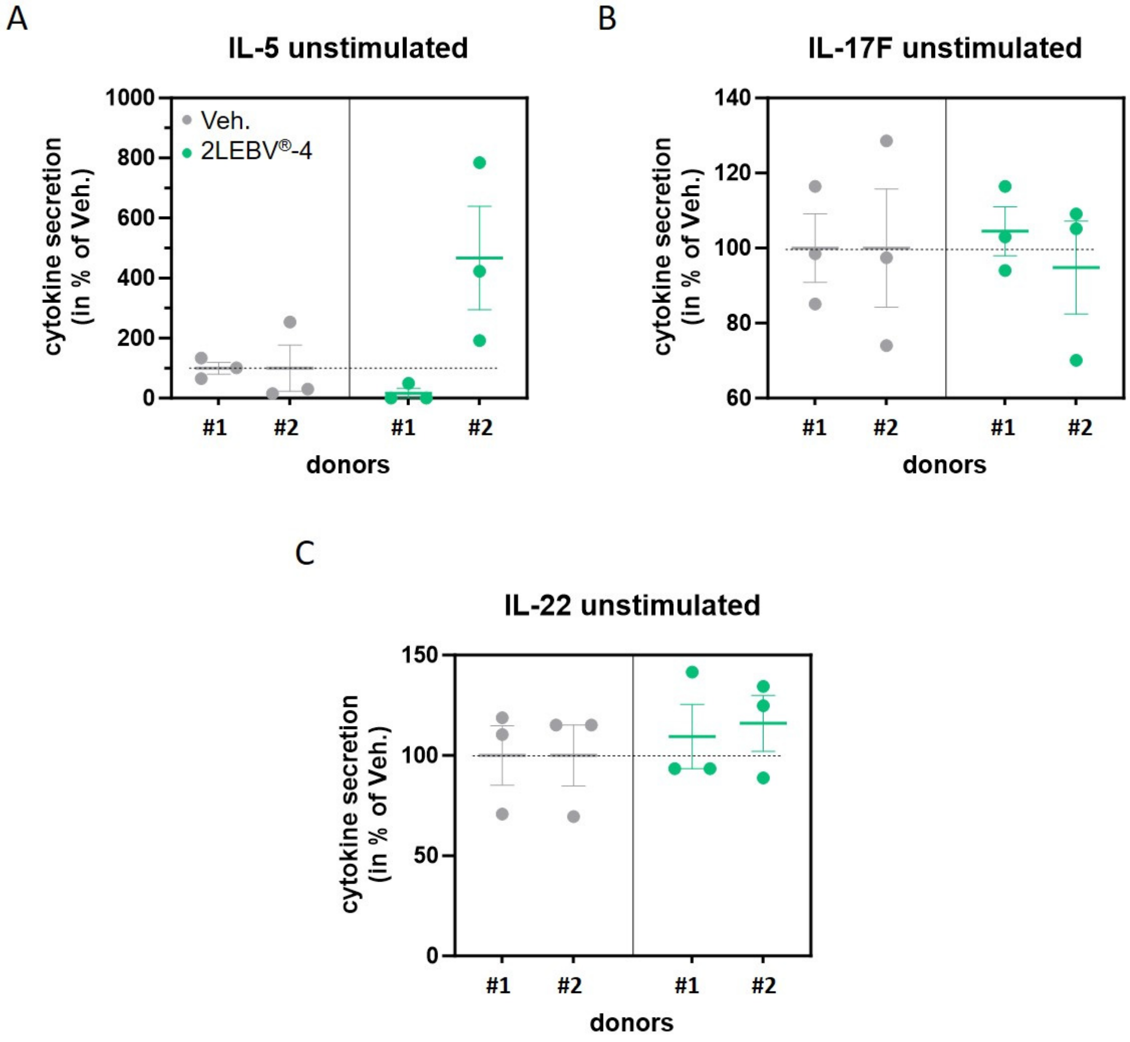

Supplementary Figure S2. 2LEBV®-4 modulates the secretion of several cytokines in unstimulated human PBMCs. PBMCs from two donors (#1 and #2), were retrieved and incubated for 48 hours in the presence of either the Veh. (grey dots), or 2LEBV®-4 (green dots), in normal unstimulated culture conditions. Thus, ELISA assay appraised the secretion of IL-5, IL-17F and IL-22 in the SN. The results are presented as the mean ± S.E.M. of n = 3 measures for each donor and expressed as a percentage of the values obtained in the Veh.-treated conditions (set as 100%). The dotted lines are drawn to highlight the effect of 2LEBV®-4 compared with the Veh.

Supplementary Table S1: Effect of 2LEBV®-4 on cytokine secretion, appraised in unstimulated or in concanavalin A (Con-A)-stimulated human peripheral blood mononuclear cells (PBMCs).

Cytokines	Stimulatory (↑) or inhibitory (↓) effects of 2LEBV®-4 on:	
	unstimulated PBMCs	Con-A stimulated PBMCs
IL-4	↑	↑
IL-5	-	↓
IL-6	↑	↑
IL-9	↓	↓
IL-17F	-	↓
IL-22	-	↓
TNF-α	↑	↑

Con-A: concanavalin A; IL: interleukin; PBMCs: peripheral blood mononuclear cells; TNF-α: tumor necrosis factor α.
